# Fibre Intake in Chronic Kidney Disease: What Fibre Should We Recommend?

**DOI:** 10.3390/nu14204419

**Published:** 2022-10-21

**Authors:** Secundino Cigarrán Guldris, Juan Antonio Latorre Catalá, Ana Sanjurjo Amado, Nicolás Menéndez Granados, Eva Piñeiro Varela

**Affiliations:** 1 Nephrology Service, Hospital Publico da Mariña, E-27880 Burela, Spain; 2 Nephrology Research Unit, Hospital Publico da Mariña, E-27880 Burela, Spain

**Keywords:** chronic kidney disease, nutrition, fibre consumption, vegetables, cardiovascular risk, microbiota

## Abstract

Chronic kidney disease (CKD) is a major global health problem that challenges all patients’ healthcare needs. Fibre consumption benefits kidney patients by acting preventively on associated risk factors, improving intestinal microbiota composition or reducing metabolic acidosis and inflammation. In this review, we focus on increasing fibre consumption and the quality of fibre to recommend, in addition to increasing the consumption of foods that naturally have it in their design, that can resort to fortified foods or fibre supplements. The Western nutritional practice, which is low in fibre and rich in animal proteins, saturated fats, sodium, and sugar, increases the risk of mortality in these patients. On the contrary, patterns with higher consumption of fibre and vegetable proteins, such as the Mediterranean, vegetarian, or Plant dominant low protein diet (PLADO), seem to have a preventive effect on the associated risk factors and influence CKD progression. Until now, the use of fibre supplements has not achieved an evident impact on clinical results. Fibre-rich foods contain other nutrients that reduce cardiovascular risk. Promoting diets richer in vegetables and guaranteeing adequate energy and protein intake is a challenge for the multidisciplinary teams involved in the standard of care for CKD.

## 1. Introduction

Chronic kidney disease (CKD) is a major global health problem that challenges all the health care these patients need [1]. Although renal deterioration can be due to primary kidney diseases, other pathologies such as obesity, diabetes mellitus, and arterial hypertension are risk factors for this disease [2]. Bearing that the number of people with obesity, arterial hypertension and diabetes mellitus, primarily type 2, is increasing and occurs in increasingly younger patients, it seems logical that the number of patients with CKD will be more significant [3].

Scientific evidence supports the importance of nutritional treatment in preventing and treating CKD [4,5,6,7].

The approach to nutrition in this disease has changed in recent years, moving from very restrictive diets to precision nutrition, seeking a correct intake of macro and micronutrients that improve the patient nutritional status. It continues to be a priority to control the dietary intake of proteins, phosphorus, sodium and potassium, providing adequate energy. Still, attention is also paid to other nutrients, such as the type of fat ingested or the contribution of antioxidants [4,5,6,7]. Increasing attention is paid to modulating the intestinal microbiota by modifying the composition of the diet to incorporate a more significant number of plant foods [6].

This increased consumption of plant foods can influence not only the development of the disease but also its prevention since it can help reduce blood pressure, improve glycemic control in diabetes patients and reduce obesity. One of the ingredients in vegetables to which these effects are due in fibre [4,7,8].

For all of these reasons, it is convenient to analyse the advantages that fibre can bring to kidney patients and what would be the most convenient way to increase dietary fibre intake.

## 2. Fibre: Definition and Sources

According to the European Food Safety Authority (EFSA), dietary fibres are carbohydrates not digestible by the digestive tract enzymes that pass intact to the large intestine and are beneficial for health [9].

All vegetables contain fibre: whole grains, nuts and seeds, legumes, fruits, vegetables and greens.

On the other hand, different fibres can be isolated from vegetables and added to foods, thus creating fibre-enriched foods such as slices of bread, cereal bars, yoghurts, and cookies.

Isolated fibre can also be used as a dietary supplement.

## 3. Recommended Dose

The EFSA recommended dose for adults is 25 g/day, which is in line with most current fibre recommendations for adults, 25–38 g/day [8].

Most of the current clinical practice guidelines for kidney patients (such as KDIGO, KDOQI, and KHACARI) do not include specific recommendations for fibre because generalising the recommendations without taking into account the characteristics of each patient could lead to some cases of hyperkalaemia [10]. Including fibre-rich foods in the diet is recommended, with a higher intake of fruits and vegetables [5].

Some authors, such as Kalantar et al., suggest a dietary fibre intake of at least 25–30 g/day to obtain all the benefits that fibre can provide [4].

From the consulted literature [7,8,11,12] and our clinical experience (Cigarran et al. data not published), average consumption is less than 16 g/day, far from the allowance values.

## 4. Fibre Types, Classification

The fibre is classified considering its physicochemical properties: fermentability, solubility in water and viscosity.

These properties determine fibre’s functional characteristics; thus, soluble fibres are the most fermentable and have a modulating action on the microbiota. The insoluble ones capture less water and are less fermentable, increase the volume of faeces, improve intestinal transit and prevent constipation. Some soluble fibres thicken when mixed with liquids due to their viscosity and decrease appetite, delay gastric emptying, slow down the degradation/adsorption of some nutrients, decrease postprandial blood insulin and glucose concentrations, improve glycemic response, and can reduce cholesterol (only demonstrated with beta-glucan and psyllium) [13,14].

On the other hand, total fibre is the sum of dietary fibre (the edible, indigestible components of carbohydrates and lignin in plant foods) and functional fibre (isolated, extracted, or synthetic fibre that has proven health benefits) [15].

Table 1 shows dietary fibre’s classification, considering its composition and physicochemical properties [13,16,17].

Bacteria feed on fermentable soluble fibres but can also use some insoluble fibres that are partially fermentable [18].

Most studies on dietary fibre in kidney patients have been performed with highly fermentable soluble fibre [19].

## 5. Benefits of Fibre for Chronic Kidney Patients

### 5.1. Preventive Effect, Acting on CKD Risk Factors

- Improves blood pressure. Several mechanisms may explain this action, such as modifying arterial contraction due to its effect on the smooth muscle of the artery, influencing the activity of the angiotensin-converting enzyme (ACE) or retaining minerals such as potassium and magnesium in its matrix [5,20].
- Improves glycemic control. In diabetic patients, fibre can delay gastric emptying, reduce glucose absorption after meals, give a lower glycemic response, produce greater satiety, and improve insulin sensitivity. Diabetes mellitus is one of the leading causes of CKD, and improving glycemic control is a fundamental goal in these patients [21,22].
- Improves the lipid profile. Soluble fibres with high viscosity decrease cholesterol absorption and can bind to bile acids, increasing their faecal excretion. Bacterial fermentation in the colon produces short-chain fatty acids that can inhibit cholesterol production in the liver. As a result, total and LDL cholesterol are reduced [18].
- Improves weight control Some factors that can influence weight loss are: increased satiety, helping to reduce energy intake, the slower absorption of some nutrients in the intestine, helping reduce inflammation, and improving constipation. Current guidelines recommend increasing fruit and vegetable intake at stages 1–4 to decrease body weight [5].

### 5.2. Changes in the Composition of the Intestinal Microbiota

Dietary fibre can change colonic microbial activity from a proteolytic (fermenting amino acid) to a saccharolytic (fermenting complex carbohydrate) fermentation pattern [6,19,23].

Dietary habits affect the composition of the gut microbiota. Since the microbiota is in contact with a significant number of neural and immunological cells, it directs the maturation of the immune system in childhood. It contributes to the maintenance of its homeostasis during life. Functionally, the gut microbiota provides nutrients and energy to the body through the fermentation of nondigestible foods in the large intestine. The most important fermentation products deriving from the fermentation are the SCFAs, which serve as a source of energy to intestinal cells and bacteria and contribute to energy expenditure, satiety, and glucose homeostasis [24,25].

A high-fibre diet increases the production of short-chain fatty acids (SCFAs), which provide energy to the intestinal flora and allow amino acids that reach the colon to be incorporated into bacterial proteins and be excreted instead of being fermented into uremic solutes. In addition, SCFAs are used as a substrate by the intestinal mucosa, helping to maintain their functionality and integrity. Fibre increases intestinal transit, reducing the time for amino acid fermentation, and improves microflora’s composition, reducing the production of undesirable solutes. In CKD patients, there is a direct relationship between dietary protein/fibre ratio and PCS and IS levels, so a diet with a low protein/fibre ratio should be beneficial. In healthy subjects, a vegetarian diet, compared with the omnivore diet, reduces the generation of IS or PCS; this effect was related to the vegetarian diet’s higher fibre and lower protein content. A very low protein diet (0.3 g/kg weight/day) supplemented with amino acid keto-analogues also reduces IS levels in patients with CKD [24,25].

The action of saccharolytic bacteria produces gases such as methane and carbon dioxide and increases the production of SCFAs, mainly acetate, propionate, and butyrate. Proteolytic bacteria produce toxic metabolites such as p-cresyl sulfate (PCS), trimethylamine n-oxide (TMAO), and indoxyl sulfate (IS). TMAO is removed in dialysis, but not IS and PCS, which bind to albumin and increase their concentrations in plasma [6,19].

SCFAs, especially butyrate, can influence the integrity of the intestinal barrier by reducing the passage of uremic toxins from the intestine to the blood, modulating the immune system and the inflammatory response through the regulation of T cells. Higher fibre intake may offset the effects of protein consumption on the progression of kidney disease [6,19,23].

The protein-fibre ratio is the ratio of total protein to total fibre; changes in the diet towards a lower protein-fibre ratio can help reduce IS and PCS serum levels [26].

Fibre intake lowers serum urea levels by providing a faecal route of excretion for accumulated nitrogenous waste. In addition, bacterial creatinase can degrade creatinine, a by-product of metabolism, in the intestine [12,19,26].

### 5.3. Effect on Inflammation and Oxidative Stress

Current data suggest that dietary fibre intake independently correlates with inflammation and oxidative stress.

The toxic metabolites of PCS and IS proteins have a proinflammatory effect and cause oxidative stress. These two uremic toxins are the most studied for their cardiovascular toxicity [6,24].

Patients with CKD have high levels of AGEs because their renal excretion is reduced, and endogenous formation can increase due to oxidative stress and, in diabetes mellitus patients, derive from hyperglycemia [27].

Fibre intake can reduce serum levels of AGE and pulse wave velocity (pwv), a non-invasive measure of arterial stiffness, and this reduction could help prevent cardiovascular events [28].

Fibre also acts as a multifactorial effect on inflammation; thus, higher fibre intake has been associated with higher plasma levels of anti-inflammatory adiponectin and lower levels of interleukin-6 and C-reactive protein (CRP). According to the results of the National Health and Nutrition Examination Survey (NHANES III), with 14,533 participants, a high fibre intake reduces CRP levels much more markedly in kidney patients than in the rest (38% for every 10 g/day increase in total fibre intake versus 11% in people without kidney disease). Furthermore, in the CKD population, higher fibre intake was associated with lower mortality, whereas in people without kidney disease, it could not be associated with mortality [29].

The ULSAM cohort study, conducted in Sweden with 1110 men aged 70 to 71 years, also indicates that CRP and IL-6 levels decreased with higher dietary fibre intake and that lower fibre intake was more strongly associated with mortality in the elderly with kidney disease than in those without it [30].

This inflammation-reducing and protective effect against adverse cardiovascular events (MACE) also have been observed in dialysis patients [31].

### 5.4. Reduced Metabolic Acidosis

Chronic metabolic acidosis is a leading complication of CKD and is associated with increased inflammation, bone disorders, hyperkalemia, insulin resistance and muscle mass loss. The acidotic state is aggravated by the vegetable restrictions to which these patients have traditionally been subjected. Pathophysiology of the acidotic condition is out of the scope of this review, and a relevant update was published in this journal in 2021 [32].

Implementing a dietetic nutritional counsel for CKD metabolic acidosis management has ample benefits, such as lipid profile control and increased vitamin and antioxidant intake [32]. With CKD progression, the mortality risk increases, and it is essential to find effective ways of controlling this condition. For this reason, counteracting metabolic acidosis helps to preserve muscle mass, avoid sarcopenia, and to improve bone metabolism [32].

Animal-based foods increase the acid load of the diet. A diet rich in plant-based foods could help control metabolic acidosis, even similar to the administration of sodium bicarbonate [33].

This buffering effect of vegetables is due, in part, to their potassium content [6]. Metabolic acidosis increases protein catabolism in muscle tissue, which prevents adaptation to a low-protein diet [6,32].

Increasing fruit and vegetable intake is recommended in adults with CKD 1–4 to reduce net acid production (NEAP) and renal acid load [5,32,34].

### 5.5. Laxative Effect

High fibre consumption is essential to reduce intestinal transit time; slow-fermenting viscous fibres retain water and soften stool. Insoluble fibres increase stool volume by stimulating intestinal transit. Fibre also promotes the growth of beneficial microbiota, improving the gut barrier, decreasing inflammation, and decreasing uremic toxin production [10].

Despite this, most people with CKD consume less than the recommended dietary fibre intake, partly due to the competing dietary potassium concern. Based on the evidence, improved information on fibre-rich food, and incorporating a multidisciplinary team, the cooperation of a dietitian is recommended to ensure a good diet plan [10].

Regular low-fibre diets, medications such as phosphate binders, iron supplements or antidepressants, and intestinal dysbiosis are reasons for the high prevalence of constipation in patients with CKD. Constipation can lead to increased retention of uremic toxins and hyperkalemia.

Dietary potassium is constantly absorbed in the intestine; however, in kidney patients, this absorption increases due to reduced renal excretion [35].

## 6. Contribution of Nutrients. Influence on the Composition of the Diet

Fibre-rich foods also have other substances that can improve the nutritional design of the diet:

### 6.1. Regarding Macronutrients

- Carbohydrates: High-fibre foods are rich in complex carbohydrates, providing a healthy energy source and a suitable substrate for saccharolytic fermentation [19].
- Lipids: By increasing the consumption of vegetables, the consumption of saturated and trans fats decreases and that of monounsaturated and polyunsaturated fats increases. Vegetable fats (except coconut oil) have a more favourable lipid profile for preventing cardiovascular events [36].
- Proteins: The ratio between the amount of protein and fibre ingested can increase the risk of kidney disease because both nutrients have opposite effects on cardiometabolic risk factors. Hence, an excess of protein in the diet concerning the intake of fibre increases cardiovascular events in these patients [26,37].

In addition to protein restriction, increased consumption of vegetable protein may influence the progression of kidney disease [7]. Although vegetable proteins have less bioavailability than animal proteins, they provide less net acid load. In addition, the phosphorus of vegetable proteins has a lower bioavailability (20–40%) because it is found as a phytate; when ingested with fibre, less phosphorus absorption also occurs. This effect is essential for reducing high phosphate levels associated with vascular calcification and cardiovascular disease [38,39].

According to recent studies, phytate itself, considered until now an antinutrient, may have antioxidant effects and the ability to inhibit the formation of vascular calcifications [40].

On the other hand, animal protein is related to induced hyperfiltration, lower insulin sensitivity, and increased reactive oxygen species (ROS). The same dose of vegetable protein does not produce the same effects [26]. Vegetable proteins can also cause a more significant decrease in blood pressure than animal proteins, according to the INTERMAP study on micronutrients and macronutrients in blood pressure [41].

### 6.2. Regarding Micronutrients

Foods with high fibre content contribute to antioxidant micronutrients such as vitamin C, tocopherols, selenium and zinc [41].

Because they are rich in almost all vitamins, except vitamins B12 and D, in addition to their antioxidant action, they have other advantages for these patients.

Vitamin K deficiency can increase cardiovascular risk and produce vascular calcification. Folates also help reduce cardiovascular risk [18].

Another abundant mineral in foods with a high fibre content is magnesium, which reduces vessel calcification and the risk of bone-mineral disorders, in addition to its action in controlling blood pressure and preventing cardiovascular risk [7].

### 6.3. Concerning Other Components

Plant-based foods provide many phytochemicals that are not nutrients, such as carotenoids and polyphenols. Although their mechanisms of action are unclear, they are known to be antioxidants and modulate oxidative stress and inflammation. This modulation is more intense in individuals with a higher level of oxidative stress and an inflammatory state. Furthermore, this effect is reduced when the different phytonutrients are used in isolation [42].

On the other hand, in large observational studies, it has been seen that the consumption of polyphenols is associated with reducing cardiovascular diseases [43,44].

Evidence suggests that nutritional interventions carried out with supplements rich in polyphenols, such as powdered grape juice or standardised pomegranate juice (or with pomegranate extract), turmeric or cocoa flavonols, could help control oxidative stress and improve inflammation in patients with end-stage kidney disease [45].

## 7. Evidence of These Effects on Actual Kidney Function

Although there is evidence that increasing fibre intake significantly reduces serum urea and creatinine [19], it is not clear that this harms the progression of kidney disease. Most studies conducted to verify this are observational, based on dietary records or fibre supplementation carried out with a small number of participants in a short time.

In a trial conducted with data from 3787 participants in the Doetinchem cohort study, consumption of whole grains, fruits, and vegetables was not associated with changes in the albumin/creatinine ratio or estimated glomerular filtration rate [46].

However, cardiovascular diseases are a common cause of death in kidney patients; arterial hypertension, hyperglycemia, hyperlipidemia and obesity are risk factors for cardiovascular diseases and CKD. These four risk factors show that dietary fibre can positively influence [16].

On the other hand, it has been seen that interventions to increase the intake of fruits, vegetables, legumes, fish, and whole grains and reduce the consumption of red meat, sodium and sugar could reduce mortality in CKD patients [47,48].

As seen in the study by Krishnamurthy VM et al., fibre intake can reduce inflammation in all patients, but the reduction in mortality is only associated with kidney patients [29].

It should be noted that fibre intake is usually assessed and not the protein/fibre ratio. However, it is known that a diet with a lower protein/fibre ratio could be more beneficial than interventions based solely on increased fibre or protein restriction [26,37].

## 8. Recommendations to Increase Fibre Consumption

To increase fibre consumption, one can resort to foods that naturally have it in their composition and use fibre-enriched foods or fibre supplements isolated from the cellular matrix.

Using a dietary fibre-based diet may be interesting, and its relationship with kidney disease has been studied.

### 8.1. Increased Consumption of Individual High-Fibre Foods

The most significant fibre consumed in Europe comes from cereals, with bread being the primary source. In second place are vegetables, potatoes and fruits, with potatoes being the most consumed in northern Europe and southern countries and fruit being the second most important dietary source of fibre [16], as shown in Table 2.

Whole grains are richer in insoluble than soluble fibre, but not those that contain white flour. They have all the fibre except pectins, especially oats and rye, which are rich in beta-glucan [16].

Vegetables are rich in cellulose and hemicellulose; insoluble fibre predominates and does not contain beta-glucans, resistant oligosaccharides, or resistant starch. Fruits are similar to vegetables but richer in pectin, and green bananas are rich in resistant starch. Potatoes and other root vegetables have the same amount of soluble and insoluble fibre, contain resistant starch, cellulose, hemicellulose, and pectin and do not contain beta-glucan or resistant oligosaccharides.

Legumes are foods with high fibre content, predominantly insoluble; they are the richest in resistant oligosaccharides and starch. Additionally, they contain cellulose and are rich in hemicellulose; they do not contain beta-glucan or pectin. Nuts and seeds are a high-fibre source, with insoluble fibre predominating. Rich in hemicellulose, they also contain pectin and cellulose. In vegetables without skin, the content of insoluble fibre is reduced [16].

If we take into account the frequency of consumption recommended by the Mediterranean diet, the only dietary pattern stated in the latest update of the KDOQI guidelines [5], we could suppose that a general distribution of vegetables is necessary to customise it for each patient, which could be:

Legumes: 2 or more times a week; Fruits and vegetables: 2 servings a day or more; Nuts and seeds: 1–2 servings a day; Potatoes: 3 servings or less a week; Cereals: 1–2 servings at each meal [5,47].

As these foods are rich in potassium, hyperkalaemia must be avoided in patients at risk of presenting it. For this reason, vegetables with a low potassium content can be used, or they can even be classified by the relationship between potassium and fibre content, giving priority to foods with a low potassium/fibre ratio [49,50,51].

Offer healthy culinary recommendations promoting increased fruit and vegetable, fish, legume, whole grain, and fibre intake to reduce red meat, sodium, and refined sugar intake. Education in cooking is highly recommended to avoid hidden sources of potassium [10].

However, it must be taken into account that all foods contain potassium and that their bioavailability in vegetable foods is lower; this could be an advantage for patients to consume these foods rich in fibre, taking advantage of their properties [7].

### 8.2. Fortified Foods/Supplements

Isolated fibre from vegetables can be added to foods to produce fibre-enriched foods, and these foods can help meet fibre recommendations to achieve beneficial health effects [19]. The availability of these products in the market is increasing in diverse foods such as fruit drinks, dairy products, instant cereals, bread, soups, snacks and bars.

The amount of fibre that could be added to food is variable; in the European Union, in the labelling of a food, it can say that it is a source of fibre, but it must contain at least 3 g of fibre for every 100 g of food. To be considered a food with high fibre content, it must have at least 6 g/100 g of food [16].

Soluble fibres are easily added to foods because they dissolve in water without causing changes in taste and texture. They are the ones that are added to drinks (juices, soups) and foods with a high water value, such as yoghurt. Inulin, hydrolysed guar gum, soy fibre, and dextrins are widely used soluble fibres [8].

Viscous fibres such as beta-glucan or mucilages change the texture of foods and are less frequently found in fortified foods. They are found in some dry foods, such as beta-glucan-rich oat bran cookies or psyllium-hulled bread, and are most commonly used as supplements.

Insoluble fibres are not added to beverages or foods with high water content; they are often added to baked goods such as bread, crackers, pea shells, or wheat bran [8].

Viscous fibres such as beta-glucan or mucilages change the texture of foods and are less frequently found in fortified foods than some dry foods; oat bran cookies or psyllium-hulled bread are most commonly used as supplements.

In some studies carried out with this type of food, it has been seen that it is not enough to add fibre but that the formulation of the product must be adequate for the purpose sought. In another study with protein-free bread and pasta enriched with psyllium and inulin [52], the added soluble fibre did not significantly influence the glycemic index; it seems that the degree of processing is more important than the addition of fibre.

On the other hand, it has been seen that they can have positive effects due to the increase in fibre, such as improving intestinal transit, reducing serum creatinine levels or reducing p-cresol by up to 37% in participants with high compliance [23,53]. Despite these data, it has not been possible to establish significant clinical repercussions in these patients [10,27,54].

Foods with different fibre types can be chosen depending on the effect sought. Foods with viscous or insoluble fibre for constipation will be selected to reduce cholesterol and blood glucose.

These fibre-enriched foods may contain significant amounts of sodium, phosphate, or potassium and may not be suitable for all kidney patients, especially those with advanced-stage disease.

Regarding the use of supplements to increase a specific type of fibre, such as oligosaccharides, beta-glucan, and mucilages for a particular purpose, they also do not contain phosphorus or potassium. Many fibre supplements are available in different pharmaceutical forms: powder, capsules, and chewable tablets.

Several studies have been carried out for kidney patients, mainly with supplements of fermentable fibre with a prebiotic effect, such as oligosaccharides, β-glucans, gums, hemicelluloses and some resistant starches, the latter being those with the most remarkable trophic effects on the mucosa of the colon. It has been shown that they can significantly reduce urea, creatinine and uremic toxins in patients with CKD [10,54].

Despite these effects, no evident impact has been observed on clinical results [10,27,54].

Factors such as the type of fibre, dose or duration of supplementation may be essential, and there is still a lack of information on use in clinical practice [10].

There is evidence that dialysis patients consume less fibre than other CKD patients, and it is possible that fibre supplementation could help achieve adequate levels. In a randomised controlled trial in hemodialysis patients supplemented for k6 weeks with a fermentable soluble fibre, it was seen that the levels of interleukin-6, interleukin-8 and C-reactive protein decreased [55].

Foods with dietary fibre, unlike supplements, are rich in other nutrients such as antioxidants, vitamins or minerals that can synergistically enhance the effects of fibre.

### 8.3. High-Fibre Dietary Patterns

There is growing interest in studying dietary patterns that can be applied to kidney disease rather than just looking at individual foods. This approach focuses on providing the right amount of all nutrients and not just restricting protein, thus producing other benefits such as improving hyperlipidemia, controlling blood glucose, and reducing weight or blood pressure [21].

Dietary patterns that increase fibre intake and reduce protein content and refined and processed foods may interest these patients. Research focuses primarily on the Mediterranean diet [56,57,58,59], the Dash diet [60,61,62], and vegetarian or vegan diets [63,64,65].

Most studies have found favourable results, especially as a preventive strategy. For advanced stages of kidney failure, it may be necessary to formulate renal diets with controlled sodium, potassium, phosphorus, and protein intake. On the other hand, it is known that the current Western dietary pattern, poor in fibre, hypercaloric, rich in animal proteins, fats (especially saturated), sodium and sugar; is associated with numerous chronic diseases such as CKD [10,18,21] and increases the risk of mortality in these patients [66].

The food distribution in the Mediterranean diet, in addition to the vegetables already mentioned, includes dairy products: 2 servings per day; eggs: 3–4 units per week; white meat: 2 servings per week, and fish/seafood: 2 or more servings per week; red meat: less than 2 servings a week, and always with fresh and unprocessed products [47].

The Dash diet is close to 3 servings of low-fat dairy products per day, and the consumption of fish and white meat [67] is also slightly higher since it is prescribed every day; this makes it richer in protein, calcium and phosphorus [62]. It could be the least suitable of the three for kidney patients, although it is effective at a preventive level [61].

Another advantage of the Mediterranean diet is the consumption of olive oil (specifically virgin extra) since it can be associated with a low risk of coronary heart disease and stroke, as well as improvements in inflammatory biomarkers in the lipid profile and with favourable effects on hypertension or insulin sensitivity. Its use is recommended as the only fat for cooking: 1–2 servings at each meal (approx. 40 mL/day) [47,54,68].

Regarding the vegetarian diet, lower excretion of PCS has been reported [63] in the healthy population with a vegetarian diet; this reduction has also been seen in kidney patients on hemodialysis [64].

This could be due to higher fibre and lower protein intake in people eating a vegetarian diet. On the other hand, due to the lower bioavailability of plant proteins, a vegetarian diet could increase the risk of protein-energy malnutrition.

The benefit of these diets is that they take advantage of the synergistic multifactorial interactions of phytochemicals present in foods of plant origin [22,65], although, especially in vegans, possible deficits of vitamins B_12_ and D, minerals such as calcium, iron and zinc, in addition to omega-3 content, should be monitored [69].

We summarise the different reviews on the effects of fibre in chronic kidney disease in Table 3.

These three dietary patterns have high potassium quantities, which should be adjusted to maintain potassium levels within the normal range, as indicated by the KDOQI 2020 guide.

A dietary pattern that has been designed for the kidney patient is [4,22,70].:

Low protein diet predominantly of vegetables. Plant dominant low protein diet (PLADO). It is a low-protein diet (0.6–0.8 g/kg/day) with at least 50% plant-based protein, based on unprocessed foods, a low sodium intake of <3 g/day, fibre from 25 to 30 g/day and caloric intake of 30–35 Kcal/kg/day.

The principal risks of this diet are protein-energy wasting (PEW), sarcopenia and hyperkalemia, although there is little evidence of these side effects. According to these authors [4,22,70], this diet could be safely recommended in patients in the early and more advanced stages of the disease and diabetic or no-diabetic patients.

To successfully implement this diet, dieticians trained in CKD must monitor the patient and ensure that dietary recommendations are met.

Although this plan is designed specifically for kidney patients, there is a lack of evidence to support its widespread use [4,22,70].

## 9. Conclusions

Fibre consumption benefits kidney patients by acting preventively on associated risk factors, improving intestinal microbiota composition or reducing metabolic acidosis and inflammation. To increase fibre consumption, in addition to increasing the consumption of foods that naturally have it in their design, one can resort to fortified foods or fibre supplements. The study of dietary patterns with different fibre content is interesting to assess their influence on kidney patients:

The Western nutritional practice, low in fibre and rich in animal proteins, saturated fats, sodium, and sugar, increases the risk of mortality in these patients. On the contrary, patterns with higher consumption of fibre and vegetable proteins, such as the Mediterranean diet, the vegetarian diet or the PLADO diet, seem to have a preventive effect on the associated risk factors and influence the progression of the disease.

Until now, the use of fibre supplements has not achieved an evident impact on clinical results. Fibre-rich foods contain other nutrients that improve the nutritional composition of the diet and influence kidney patient outcomes. Finding a way to reconcile a diet richer in vegetables with the restrictions of these patients, such as potassium control, and guaranteeing adequate energy and protein intake is a challenge for the multidisciplinary team in the standard of care for kidney patients.

**Figure 1:**
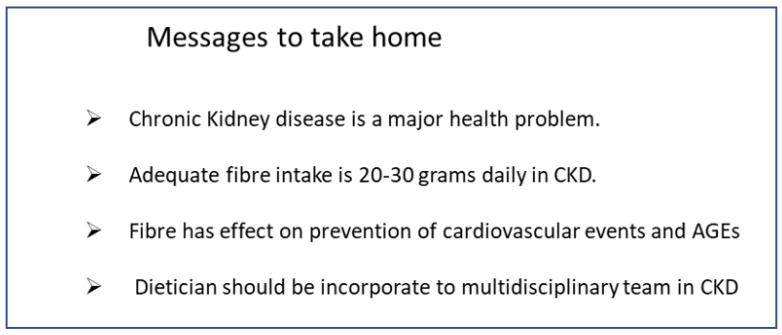
Key points to resume fibre use.

Finally, we resume in Figure 1 the key points to take into account:

## Figures and Tables

**Table 1:** Classification of dietary fibre.

**SHORT CHAIN CARBOHYDRATES**
Resistant oligosaccharides FOS and GOS. They are soluble and highly fermentable.
**LONG-CHAIN CARBOHYDRATES**
Resistant starch (RS): Soluble and highly fermentable.
Non-starch polysaccharides (NSP):
** Soluble**
-Highly fermentable: Pectins, Inulin, dextrin, glucomannan and gums (such as guar gum).
-Partially fermentable: mucilages such as psyllium seeds and beta-glucan.
** Insoluble**
-Wheat bran (barely fermentable).
-Cellulose and hemicellulose (non-fermentable or poorly fermentable).
**Lignin**
Insoluble and poorly fermentable.

**Table 2:** Total dietary fibre (TDF) in vegetal foods (soluble fibre-insoluble fibre). Data adapted from Stephen et al. [16].

Food Groups	TDF g/100 g	Soluble Fibre (%TDF)	Insoluble Fibre (%TDF)
Whole wheat bread	5.6–7.2	27	73
Oat porridge	1–7	52	48
Rye-based products	3.9–5.9	44	56
Vegetables	0.5–6	37	63
Fruits	0.4–10.4	43	57
Nuts and seeds	1.3–14.4	32	68
Legumes	4.2–10.6	25	75
Potatoes	0.5–8	48	52

**Table 3:** Summary of relevant trials of fibre with beneficial effects.

Author/s	Year	Aims	Results
Kalantar-Zadeh K et al. [4]	2020	A low-protein diet with a predominance of vegetables (>50% plant-based protein, PLADO) Plant-Dominant Low Protein-Diet.	PLADO diet administered by a specialised CKD dietician may improve prevention or delay the start of dialysis.
Carrero JJ et al. [7]	2020	To review the effects of plant-based diets in people with chronic kidney disease.	With adequate dietary advice, plant-based diets could benefit patients with CKD, such as increased fibre, changing intestinal microbiota profile, and reduced production of uremic toxins or metabolic acidosis.
Su G et al. [10]	2021	Review on the benefits of fibre to improve the progression of chronic kidney disease	High fibre intake regulates the intestine, favours healthy bacteria growth, and improves the intestinal barrier effect.
Chiavaroli et al. [19]	2014	Systematic review and meta-analysis of the effect of dietary fibre intake on urea and creatinine as markers of kidney health in patients with CKD.	Demonstrates the potential beneficial effects of dietary fibre on the reduction of serum urea and creatinine.
Salmean et al. [23]	2015	To determine the effects of a fibre supplement, in a single-blind supplement, on plasma p-cresol production, stool frequency and quality of life (QoL) in patients with chronic kidney disease.	The production of p-cresol decreases, and the frequency of bowel movements increases; no changes are observed in the general quality of life.
Rossi et al. [26]	2015	To conduct a randomised controlled trial of synbiotic therapy in patients with CKD to study the influence of fibre intake on the generation of IS and PCS toxins.	The dietary protein-fibre ratio is associated with serum levels of IS toxins and PCS rather than with each of them individually.
Demirci BG et al. [28]	2019	To analyse the relationship between the effect of total dietary fibre intake on C-reactive protein (CRP) and oxidative stress parameters, such as advanced glycation products (AGE) in serum, superoxide dismutase (SOD), malondialdehyde and arterial stiffness by pulse wave velocity (PWv) in a patient on maintenance hemodialysis.	Adequate fibre intake could prevent cardiovascular events and inflammatory processes in patients undergoing maintenance hemodialysis.
Krishnamurthy VM et al. [29]	2012	To determine if fibre intake is associated with decreased inflammation and mortality. The National Health and Nutrition Examination Survey III has a 5.8% prevalence of kidney disease.	In the population without chronic kidney disease, higher fibre intake was associated with lower inflammation but not with lower mortality. People with chronic kidney disease were associated with less inflammation and lower mortality.
Goraya N et al. [33]	2014	To analyse the treatment of metabolic acidosis in patients with chronic kidney disease, stage 3, with oral bicarbonate or ingestion of fruits and vegetables in a randomised but unblinded trial.	At a 3-year follow-up, fruit and vegetable intake reduced renal angiotensin II activity and maintained a glomerular filtration rate similar to bicarbonate administration.
Xu H et al. [37]	2016	Demonstrate that CVD events in CKD may be associated with dietary patterns aligned with excess dietary protein relative to fibre. A prospective cohort study from the Uppsala Longitudinal Study of Adult Men.	A high protein intake relative to fibre intake was more strongly and independently associated with the incidence of CVD events. In isolation, fibre or protein intake was not significantly related to cardiovascular events.
Buades Fuster JM et al. [40]	2017	To review the role of plant-based phosphates in patients with chronic kidney disease.	The consumption of plant foods increases blood phosphorus levels and provides fibre and phytic acid that can reduce vascular calcifications.
Kelly et al. [48]	2017	A systematic review assesses the association between dietary patterns and mortality among adults with CKD.	Healthy dietary patterns (a higher intake of fruits, vegetables, legumes, and fish and a reduction in the intake of red meat, sodium and refined sugar) are associated with lower mortality in people with CKD.
Yang HL [54]	2021	A meta-analysis using only randomised controlled trials (RCA) to assess the influence of fibre on uremic toxins.	Fibre supplementation can significantly reduce the levels of uremic toxins in patients with CKD, this effect being more evident in non-diabetic patients on dialysis.
Xie LM et al. [55]	2015	A randomised placebo-controlled trial that analysed the effects of dietary fibre supplementation on oxidative and inflammatory status in hemodialysis patients.	Fermentable fibre supplementation in the diet improved the lipid profile and oxidative status and decreased the systemic inflammatory status of hemodialysis patients.
